# Epidemiological

**Published:** 1890-07

**Authors:** 

**Affiliations:** Kiel, A. H. Z.


### EPIDEMIOLOGICAL.

DR. KUNKEL, OF KIEL, A. H. Z., 7, 90.

Returning in July from Wisbaden, I found a malignant epidemic of diphtheria, and the epidemic remedy—*i. e.*, that which carries the patient through *in every stage*, was Mercurius-sol. 3d c., which always sufficed with me in that disease, where others rely on different mercurial preparations, as the Cyanide, for example. Indications: Nocturnal aggravations, heat, with desire to uncover, thirst, the characteristic fœtid breath, slight coating of the gums. Heat from feather beds unbearable; they not only uncover, but the head looks for a cool spot on the pillow. Constipation or diarrhœa, both with tenesmus, the children hold on to the vessel after the stool; jumentous, foul-smelling urine; night-sweats, also of a foul odor. Such easy treatment suited for six weeks, when Mercur. ceased to be of any benefit, as the picture of the disease had changed, and the epidemic remedy was now Aurum for awhile. Indications: high fever, one hundred and four to one hundred and five, enormous swelling of the cervical glands coming on rapidly; foul, unbearable odor from the mouth; sometimes an eruption, reminding one of scarlatina; foul alvine discharges; urine saturated. Though cases were malignant enough, Aurum acted now as promptly as Mercur. did before, especially in relation to the fever, only it took a longer time to recover on account of the glandular swellings. With the Aurum cases it also was characteristic that the nasal membrane suffered from the diphtheritic process. Nose clogged up, discharging a watery, foul fluid, redness and sensitiveness of the nose. Simultaneously with the diphtheritis the usual summer diarrhœas appeared, sometimes very severe, and Veratrum-album was the remedy; copious vomiting and copious stools; coldness of the whole body, cold sweat, especially of the forehead; collapse imminent; thirst for cold drinks in large quantities, which were not well borne; scanty urination. At once

Veratrum began to fail and Apis took its place, 7th *x*. Lighter cases with more diarrhœa and some tenesmus. During the fall more sporadic cases, especially of diphtheria. In one very severe case, a child of seven years, with the necrotic form, the stench in the room was unbearable; uvula and soft palate seemed destroyed, but Secale-cornutum 3d c. removed the disease in a few days, so that the child was able to run about, with satisfactory appetite, when paralysis of the heart finished the case. In several other severe cases, though not necrotic, Secale acted well. Now influenza followed, and Kunkel could not find the epidemic remedy. The influenza became dangerous by its complications, especially diseases of the respiratory organs, pneumonia, following a bronchitis or pleurisy; in the former Phosphorus 3d, in the latter Bryonia 2d or 3d, also Arsenicum were the remedies, at the beginning of the disease, with severe headache, vomiting, deliria, photophobia, vertigo, and congestions to the head. Belladonna often cut short the disease, though many cases recovered rapidly without taking anything. Indications for Arsenic: Thirst, but little suffices; restlessness, wants the head raised up; with the dyspnœa anguish, awaking with anguish and deliria. It is a well-known fact that epidemics of influenza precede cholera, and it was remarkable that the most effective remedies in cholera (Arsenicum, Cuprum, Veratrum, Phosphorus) yielded the best results in the most severe cases of influenza. Cuprum acted especially well in pneumonia of senility and of drunkards. Cuprum in such cases may prevent cardiac paralysis, and it may be, perhaps, advisable to give Cuprum for a few days during the reconvalescence from severe influenza.

---

This indication for Cuprum ought to be verified before being incorporated. I, for one, feel glad to have another anchor to hold more firmly the fast-ebbing life in cardiac paralysis after severe zymotic diseases, for I lost several cases during reconvalescence when my warning voice was disregarded, and they succumbed in spite of Camphora, Veratrum, Carbo-veg. In the collapse of Cuprum cramps are especially prominent, so it is said, but there were none present in my cases, and uræmia could be totally excluded. They fell asleep because the weak heart had lost its propelling power; and, let us make a mark of it, that when this lack of reaction takes place in patients run down by the severity of the disease, our first duty is to keep the patient strictly in a horizontal position, even during defecation and micturition; the nurse must feed the patient, as it is done in Mitchell's treatment, and our second duty is to remember Cuprum in its action for a weakened, and often dilated heart.

